# Dropout From an eHealth Intervention for Adults With Type 2 Diabetes: A Qualitative Study

**DOI:** 10.2196/jmir.7479

**Published:** 2017-05-30

**Authors:** Silje Stangeland Lie, Bjørg Karlsen, Ellen Renate Oord, Marit Graue, Bjørg Oftedal

**Affiliations:** ^1^ Department of Health Studies University of Stavanger Stavanger Norway; ^2^ Stavanger University Hospital Stavanger Norway; ^3^ Centre for Evidence-based Practice Western Norway University of Applied Sciences Bergen Norway

**Keywords:** eHealth, Telehealth, type 2 diabetes, Internet, counseling, qualitative research, general practice, self-management, self-management support, patient dropouts

## Abstract

**Background:**

Adequate self-management is the cornerstone of type 2 diabetes treatment, as people make the majority of daily treatment measures and health decisions. The increasing prevalence of type 2 diabetes mellitus (T2DM) and the complexity of diabetes self-management demonstrate the need for innovative and effective ways to deliver self-management support. eHealth interventions are promoted worldwide and hold a great potential in future health care for people with chronic diseases such as T2DM. However, many eHealth interventions face high dropout rates. This led to our interest in the experiences of participants who dropped out of an eHealth intervention for adults with T2DM, based on the Guided Self-Determination (GSD) counseling method.

**Objective:**

In this study, we aimed to explore experiences with an eHealth intervention based on GSD in general practice from the perspective of those who dropped out and to understand their reasons for dropping out. To the best of our knowledge, no previous qualitative study has focused on participants who withdrew from an eHealth self-management support intervention for adults with T2DM.

**Methods:**

A qualitative design based on telephone interviews was used to collect data. The sample comprised 12 adults with type 2 diabetes who dropped out of an eHealth intervention. Data were collected in 2016 and subjected to qualitative content analysis.

**Results:**

We identified one overall theme: “Losing motivation for intervention participation.” This theme was illustrated by four categories related to the participants’ experiences of the eHealth intervention: (1) frustrating technology, (2) perceiving the content as irrelevant and incomprehensible, (3) choosing other activities and perspectives, and (4) lacking face-to-face encounters.

**Conclusions:**

Our findings indicate that the eHealth intervention based on GSD without face-to-face encounters with nurses reduced participants’ motivation for engagement in the intervention. To maintain motivation, our study points to the importance of combining eHealth with regular face-to-face consultations. Our study also shows that the perceived benefit of the GSD eHealth intervention intertwined with choosing to focus on other matters in complex daily lives are critical aspects in motivation for such interventions. This indicates the importance of giving potential participants tailored information about the aim, the content, and the effort needed to remain engaged in complex interventions so that eligible participants are recruited. Finally, motivation for engagement in the eHealth intervention was influenced by the technology used in this study. It seems important to facilitate more user-friendly but high-security eHealth technology. Our findings have implications for improving the eHealth intervention and to inform researchers and health care providers who are organizing eHealth interventions focusing on self-management support in order to reduce dropout rates.

## Introduction

eHealth interventions are promoted worldwide and hold a great potential in future health care for people with chronic diseases such as type 2 diabetes mellitus (T2DM). However, many eHealth interventions face adoption problems and high dropout rates [1-5]. This led to our interest in the experiences of participants who withdrew from an eHealth intervention for adults with T2DM at general practices in Norway.

Diabetes is a chronic disease affecting an estimated 415 million people worldwide. Most of them have T2DM and its prevalence is rapidly increasing [6]. People living with diabetes are recommended to engage in multiple self-care behaviors such as taking medications, following a diet, engaging in regular physical activity, and self-monitoring, in addition to problem-solving and coping [7]. These are all aspects of diabetes self-management and essential to blood glucose control for the prevention of long-term complications. Many people with T2DM find adequate self-management difficult to achieve and maintain [8]. Some of the recommended self-management behaviors do not coincide with peoples’ priorities and desire for a “normal life.” They may differ from people’s habits and preferences and be perceived as burdensome [9,10]. Research indicates that only 1 in 8 patients with T2DM achieves the recommended treatment goals of glycemic control, cholesterol, and blood pressure [11]. Consequently, to achieve adequate self-management and optimal treatment outcomes, many patients need support from a health care professional. Given the increasing prevalence of T2DM, there is a need for innovative and effective ways to deliver self-management support interventions for people with T2DM. eHealth self-management support interventions can assist people with adopting and maintaining behaviors needed for adequate diabetes self-management [12-14].

Secure messaging is an eHealth technology that facilitates personal and interactive communication between health care providers and patients. A systematic review of participatory Web-based interventions found that asynchronous communication tools such as secure messaging was experienced as particularly useful for self-management support [2]. Such communication between patients and health care providers seems to improve effects and adherence in eHealth interventions [15-17]. Moreover, previous research has addressed the need for theory-based eHealth interventions for T2DM [14]. Theory-based interventions are valuable as the theory inform intervention strategies. These strategies translate into key components of the interventions that can be applied and assessed, thus facilitating explanation of observed effects or lack thereof [18,19].

As a response to the need for effective and theory-based interventions for people with T2DM, we adapted the self-management support intervention Guided Self-Determination (GSD) for T2DM [20], as an eHealth intervention via secure messaging in general practices (Table 1 and Textbox 1). GSD is a counseling approach founded on the self-determination theory (SDT). This theory proposes that in order to foster autonomous motivation for engagement in activities, it is important to support individuals’ basic psychological needs for autonomy, relatedness, and competence [21]. The GSD intervention aims to support diabetes self-management by empowering self-determined goal-setting and competence-building [22,23]. The intervention is described in more detail in the Methods section.

Some eHealth interventions show dropout rates of up to 80% [3-5]. A systematic review, exploring Web-based interventions designed to support and promote diabetes education and health behavior change for management of T2DM, similarly shows that intervention-engagement and usage declined over time. About half of the interventions focused on support and coping skills, and the most targeted behaviors were physical exercise, diet, and blood glucose self-monitoring [15]. A meta-analysis of the effectiveness of Web-based tools for people with diabetes suggests that participants’ difficulties in understanding the use of Web-based interventions led to higher dropout rates [24]. Moreover, a study investigating adherence to a Web-based intervention to support diabetes self-management through components derived from social cognitive theory (such as modeling-videos, information, and tools to monitor own target behavior), indicates that Web-based trials should plan for a 50% dropout rate in the first month of the intervention [25]. In a 2016 study, close to every second patient did not log on more than once to a personal health record with self-management support and personal feedback for patients with T2DM. Only five of 132 participants used the eHealth self-management support program with goal setting and action planning functionality. Three out of these five took advantage of the personal feedback offered by the health psychologist [26].

Dropout and nonuse are thus major challenges in eHealth interventions, including those offering self-management support and personalized feedback. This makes it imperative to explore experiences of such interventions among people who drop out. To the best of our knowledge, no previous study has conducted qualitative interviews with participants who dropped out of an eHealth counseling intervention designed to support self-management for people with T2DM. The aim of this study was therefore to explore experiences with the eHealth intervention based on GSD from the perspectives of those who dropped out and to provide insight into their reasons.

**Table 1:** Overview of the Guided Self-Determination counseling for adults with type two diabetes and the reflection sheets.

Consultations	Focus	Reflection sheets
The first session at the GP^a^’s office	Preparing for subsequent consultations	Invitation to work together The HbA_1c_^b^measurement
eConsultation 1	Your life with diabetes	RS^c^1a. Important events and periods in your life RS 1b. At present, what do you find difficult about living with diabetes? RS 1c. Unfinished sentences – your needs, values, habits and opportunities RS 1d. A picture, metaphor or expression of your life with diabetes
eConsultation 2	Focus for change	RS 2a. Room for diabetes in your life RS 2b. Your plans for changing your way of life
eConsultation 3	Work with changes	RS 3a. Clarification of challenge in your life with diabetes RS 3b. Previous problem-solving: thoughts, feelings, goals, and actions RS 3c. Dynamic problem-solving
eConsultation 4	Changes in daily life	RS 4a. Blood glucose self-monitoring and your reasons for self-monitoring RS 4b. New strategies and long-term plan for change RS 4c. Dynamic judgment of current and future problem solving RS 4d. «Pros and cons»

^a^GP: general practitioner.

^b^HbA_1c_: glycosylated hemoglobin.

^c^RS: reflection sheet.

**The Web portal.**

The secure messaging service was provided by the portal MinJournal. The secure messaging system at the portal demands login with electronic identification (BankID), providing the highest level of security (security level 4). Norwegian law requires this for Web-based sensitive information transfer, such as asynchronous communication between patients and health care personnel. This platform is already in use in Norwegian health care.

## Methods

### Design

We used a qualitative design and collected data by means of individual telephone interviews with participants who withdrew from the GSD eHealth intervention.

### Description of the Guided Self-Determination (GSD) eHealth Intervention

General practice was chosen as an applicable intervention site because general practitioners (GPs) and registered nurses working with GPs are primarily responsible for health care for T2DM in Norway. The GSD eHealth intervention was delivered in addition to regular care. Regular care consists of structured annual consultations with a GP and nurse, as well as recommended routine measurement of glycosylated hemoglobin (HbA_1c_) and consultations with a GP every 3-4 months, or individually adapted [20,27].

The aim of the GSD intervention was to support diabetes self-management. The participants answer questions on reflection sheets, and the themes addressed are then discussed with the nurse [28]. Table 1 shows an overview of the 4 eConsultations and topics of the 13 reflections sheets used in the GSD eHealth intervention for T2DM.

In this study, 4 trained nurses experienced in diabetes care at general practices delivered the GSD eHealth intervention over 12 to 35 weeks from August 2015 to April 2016. To establish a relationship, the nurse and patients initially met face-to-face at the GPs office. The nurse explained the aim of the GSD counseling, how to work with the reflection sheets (Table 1), and how to log on to the Web portal to use the secure messaging system (Textbox 1). All patients received a manual describing how to use the portal, the process of downloading and uploading portable document formats (PDFs) to the secure messages, how to fill out the reflection sheets, and send secure messages. After this initial meeting, the patients and nurses were to conduct 4 eConsultations, each consisting of 2 to 4 message exchanges. The patients were to complete the reflection sheets belonging to each eConsultation at home on their own electronic device, using their own words to express and reflect on their experiences and difficulties with diabetes management in daily life. They also formulated goals and plans for self-management. The reflection sheets were sent to their nurses via secure messages. The purpose of the reflection sheets were to facilitate situational reflection and improve communication to enable autonomous problem-solving, goal setting, and action planning (Table 1) [23].The nurses responded with written feedback to the participants’ reflections.

### Recruitment

Overall, 18 people invited by nurses at 4 general practices in southwestern Norway agreed to participate in the GSD eHealth intervention. However, 13 of these 18 eventually left the intervention. The nurses who conducted the intervention invited the participants who had dropped out to take part in telephone interviews with a researcher. One person declined and 12 agreed.

### Data Collection

Data were collected through telephone interviews in the spring of 2016. Telephone interviews are useful for collecting qualitative data and are considered less time- and energy-consuming for participants than face-to-face interviews [29,30]. The first author performed all interviews according to a semistructured interview guide. The main question invited the participants to speak freely and was expressed this way: “What was your experience with the GSD eHealth counseling intervention?” Supplementary questions were asked during the conversation to invite clarification and elaboration. Examples were “When and why did you quit the intervention?” “What were your expectations?” and “How did you experience written communication with your nurse via secure messaging?” The interviews lasted an average of 20 min, were audiotaped, and subsequently transcribed verbatim. In addition, demographic and clinical data were collected by a questionnaire, which the participants completed at the start of the intervention.

### Data Analysis

The transcribed interviews were subjected to qualitative content analysis as described by Graneheim and Lundman [31]. All interviews were the unit of analysis and were read by 4 members of the research team at the beginning of the analysis process to attain a comprehensive understanding of the data. Meaning units responding to the aim of the study were identified and shortened but with core content preserved. The condensed meaning units were then labeled with tentative codes, after which categories were created by comparing and grouping codes according to similarities and differences. The categories were interpreted and abstracted into a main theme. Next, to strengthen the credibility of the analysis, the research team discussed and revised the codes, categories, and main theme several times until consensus was reached.

### Ethical Considerations

The Norwegian Regional Committee for Medical and Health Research Ethics (REK west No.2015/60) approved the study. All participants signed a written consent form and were guaranteed anonymity and the right to withdraw from the study at any time.

## Results

### Description of Participants

**Figure 1:**
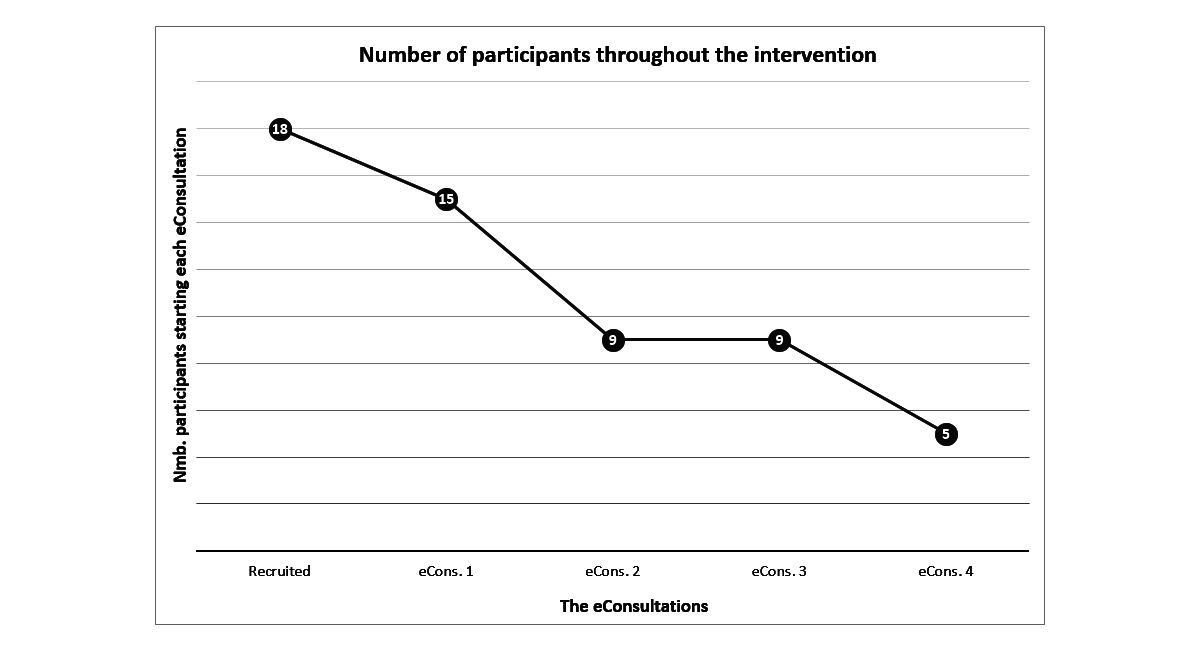
Dropout graph.

Participant characteristics are presented in Table 2. Of the 18 participants with T2DM recruited to the intervention, 14 were men and 4 were women. Of the 13 participants who dropped out, the majority (n=9) dropped out in the initial stage of the GSD eHealth intervention, before or during the first eConsultation. The last 4 participants withdrew during the third eConsultation (see Figure 1). Eleven of the 18 participants had an HbA_1c_≤ 7%, which is the expected treatment goal. The participants who dropped out from the intervention (n=13) did not differ considerably from those who completed the intervention (n=5). However, some small differences were detected; mean HbA_1c_ were 7.1% for the former and 7.7% for the latter. More men withdrew than women. All participants who regulated their diabetes with diet only withdrew from the intervention. Also, the median duration of diabetes was 9 years for those who dropped out and only 2 years for those who completed the intervention.

**Table 2:** Participant characteristics.

Demographics		All 18 participants recruited to the intervention	The 13^a^participants who dropped out of the intervention
Women (n)		4	2
Men (n)		14	11
Mean age (years, range)		55 (42-73)	57 (44-73)
Mean HbA_1c_^b^ (%, range)		7.3 (5.8-10.0)	7.1 (5.8-10.0)
Median diabetes duration (years, range)		9 (2-15)	9 (2-15)
**Living situation (n)**			
	Alone	4	3
	With family	14	10
**Educational status (n)**			
	Higher education >4 years	1	0
	Higher education <4 years	6	4
	Upper secondary education	8	6
	Primary school	3	3
**Occupational status (n)**			
	Working full-time	15	10
	Retirement pensioner	2	2
	Receiver of disability benefit	1	1
**Diabetes treatment (n)**			
	Diet	4	4
	Oral or other medications	11	7
	Insulin	3	2

^a^12 were interviewed in this study.

^b^HbA_1c_: glycosylated hemoglobin.

### Overview of Findings

The analysis resulted in identification of one theme related to experiences of the participants who dropped out of the GSD eHealth intervention: losing motivation for intervention participation. This theme described how motivation for participating in the intervention was influenced by some discouraging experiences. It was based on four categories: (1) frustrating technology, (2) perceiving the content as irrelevant and incomprehensible, (3) choosing other activities and perspectives, and (4) lacking face-to-face encounters. These categories are presented below and illustrated with quotations to facilitate transparency of interpretation. The quotations are attributed to the participants [P1-P12] to demonstrate their experiences and opinions.

#### Frustrating Technology

This category focuses on how participants felt frustrated by the technology used in this eHealth intervention. Initially, participants reported being receptive to participating in the GSD eHealth intervention. They valued the time and resource-saving potential of electronic communication with their nurse. However, they described difficulties in navigating the Web page due to errors with the portal and perceived the Web solution as time-consuming and tiring:

There was just too much trouble with it (the web page). In the end, I just gave up trying. Had it only been easier...

P12

Participants stated that it was cumbersome to download and save the PDFs before filling out the reflection sheets. They would have preferred completing the reflection sheets directly on the Web page. Participants also experienced Web page errors, for instance downtime, login problems, alerts from the firewall that it was an insecure Web page (which it was not), or that the nurse had not received the messages they sent. Some described being irritated and frustrated by technological problems. They pointed out that the Web solution bothered them when they were unable to send secure messages:

I answered the questions and tried to send, but it did not send. I tried several times, and I could not do it. This made the whole thing stressful for me...I bothered myself with it because I did not understand it and was not able to send anything. It was a bit silly, but it bothered me a lot, that I didn’t get it...I feel like those kinds of things could be manageable, those forms, sending them. So I don’t know what it was with this web page, why it didn’t work.

P2

Although most participants experienced some challenges with the Web solution, some considered the problems minor. They said having to resend undelivered messages and change the browser to access the Web page were acceptable difficulties in an eHealth intervention.

#### Perceiving the Content as Irrelevant and Incomprehensible

Some participants did not see the content of the GSD as tailored to their needs and expectations for a diabetes self-management intervention. They expressed that they lost interest after reading some of the first issues raised in the reflection sheets because they could not familiarize themselves with these issues and did not consider the content relevant to their diabetes. As one participant noted:

I felt as if some constellations were made that I could not familiarize myself with. I live a completely normal life really; it’s just the food, and the blood glucose level that makes me attend to it. But I have managed to adapt to the situation. And I keep adapting more gradually...I felt that it didn’t suit me.

P3

The participants who reached the third eConsultation worked with reflection sheets intended to stimulate people to reflect on their goals and diabetes self-management behaviors. However, the purpose of these reflection sheets was described as difficult to understand:

When I came to “dynamic problem-solving” I started losing interest. I wondered: what do you want here? What method is this? I did not understand the purpose behind the form.

P9

Moreover, some of the participants stated that they did not fully understand what the intervention entailed when they signed up for it. Three of them said that they would prefer being able to send messages in free text to their nurse on their own schedule, instead of participating in a structured counseling intervention.

#### Choosing Other Activities and Perspectives

This category concerns the participants’ narratives of more important priorities in their lives than the GSD eHealth intervention. Examples were other illnesses that needed more attention and other personal or work-related responsibilities. Daily life consisted of many complex tasks and commitments:

I am quite busy. I work full time and I really like to read. I have so much reading material, and I am active in politics as well. I have so much to read, so that just going online and having to spend much time there...It took too much of my time. Therefore, I felt it was a bit like...I didn’t like that so much. I felt it took too much time.

P11

Going on the Web and engaging in the GSD eHealth intervention seemed to be considered less important than other matters requiring their attention, and the participants therefore chose to minimize their engagement with it:

It was the required time that did it. Some of the questions also, but that was not the main reason. It was more that it became a bit too much on top of everything else, having to sit down and spend time there, and remember to send and, yeah...There was too much else that had to be paramount somehow. Therefore, I simply had to downgrade it.

P5

Choosing not to focus on diabetes was also mentioned. Being uncomfortable with the issues raised in the reflection sheets or feeling pathologized by the demanding questions were articulated. Wanting to focus on living their life illustrates this perspective:

Because I feel healthy, and I do not want to be sick. But I am sick. Therefore I do have to look after it in the long run. But there is something in my head that I can’t seem to get right...I have a diagnosis, but I do not run around being sick. I can explain some of this. My diet is what is wrong, or my life situation towards it (the diabetes). But I want to live as well. There is a limit there somewhere

P9

#### Lacking Face-to-Face Encounters

This category concerns the experience of lack of dialogue and a preference for face-to-face encounters with their nurse:

I would miss sitting down, see each other, and talk to each other. Because I’m not so into all the electronic communication. I really like to sit down and see the person I’m talking to.

P4

Meeting the nurse in person was emphasized as a motivating experience. One participant felt more obligated to try to reduce HbA_1c,_ for example, when communicating with the nurse in person. Participants also stated that answering questions verbally was easier than writing down the answers, and that they would rather speak with the nurse in their regular consultations with the nurse. The following quotation illustrates this preference:

I think it is a lot better to sit and talk with her (the nurse) right in front of me. You know, and then we can discuss things and talk a little bit like that...And if there is any misunderstanding we can ask when we’re sitting right next to each other.

P8

In addition, having eConsultations without a scheduled appointment with the nurse was considered less binding than regular health consultations:

It was allocating the time to it I had problems with...Although committing to answer, it does not have the same “disciplining” effect that one gets by meeting up at the doctor's office.

P5

At the same time, some participants emphasized that written messages could improve communication with the nurse by enabling carefully considered answers. They valued the ability to read and reflect upon the questions before answering:

The information you are able to provide about your health condition is much more thorough and better over the internet, when you sit and think through what you are going to answer and how to answer and that kind of thing. Than meeting up at the GPs office.

P12

Some of the participants insisted that they were accustomed to electronic and written communication. They appreciated the potential benefits of digital communication in health care, and some of them even preferred it, given they had the need for it. They mentioned that asynchronous digital communication could be time- and resource-saving. A combination of eHealth and regular encounters with the nurse was suggested as preferable when conducting the GSD, compared with merely written communication via secure messages.

## Discussion

### Principal Findings

This study provides insight into experiences with an eHealth intervention based on GSD from the perspective of those who dropped out and into their reasons for dropping out. Our findings indicate that the GSD eHealth intervention without face-to-face encounters influenced the participants’ motivation for the intervention negatively and resulted in dropout. Other factors that diminished their motivation pertained to choosing other activities and perspectives in their lives, perceiving the content as irrelevant, and the technology as frustrating. We discuss these findings considering earlier research and in relation to the dimensions of autonomy, relatedness, and competence proposed by the SDT as important to develop and maintain autonomous motivation.

### Comparison With Prior Work

#### Interventions With or Without Face-to-Face Encounters

Our findings indicate that participants missed face-to-face encounters with the nurse when communicating asynchronously via secure messages in the GSD eHealth intervention. They stated that they found it easier to discuss a variety of issues with the nurse and avoid misunderstandings when meeting face-to-face. Secure messages may have advantages for patient-nurse communication, such as efficient communication at convenient points of time in addition to the ability to think about the message before replying. However, our findings show the importance of acknowledging the drawbacks of written communication, such as the lack of nonverbal communication and the inability to ask immediate follow-up questions. Earlier research has demonstrated that support provided by clinicians via email enhanced adherence in eHealth interventions [32]. In contrast, our findings suggest that written communication alone is not experienced as motivating enough and that additional face-to-face encounters would have been preferred.

This could relate to the SDT, which proposes that a sense of relatedness is essential for motivation [21,33]. If people feel connected to their nurse in a warm, positive, and interpersonal manner, they may become more autonomously motivated to engage in health-related activities such as the GSD eHealth intervention [34]. Written communication via secure messages may not have been conducive to this sense of relatedness. Furthermore, we propose that our findings have some bearing on a previous study that suggests that the people with T2DM who presumably benefit the most from eHealth facilities actually use it the least [35]. This study furthermore suggests that patients’ motivation to improve T2DM self-management is not sufficiently supported by eHealth facilities. This might have been the case for some of our participants. Combining eHealth with regular consultations has been suggested by earlier research as a promising way to improve engagement and reduce attrition [26]. Some of our participants also suggested that this would improve the GSD eHealth solution.

Moreover, our findings suggest that the current eHealth intervention was seen as less important when the participants had to engage in it on their own time and had no standing appointment with the nurse. This could reflect that asynchronous Web-based health consultations are regarded as less obligatory than regular health consultations with a scheduled appointment. This adds to findings from a recent study suggesting that planning for human support and interaction could be essential to upkeep motivation and use of digital interventions [36]. eHealth combined with regular consultations may be an important topic in future research, to facilitate the personal relationship between the participants and the health care personnel needed to motivate those who truly need and could benefit from self-management support interventions.

#### Lack of Perceived Value of the Intervention

Our findings indicate that participants had commitments that required more attention than diabetes and the GSD eHealth intervention. This was illustrated by narratives of other illnesses or daily responsibilities and competing life demands that required focus and reduced their motivation for participation. According to the SDT, the value people place on various activities affects their motivation [33]. Autonomous motivation is supported if people identify with behaviors or tasks, or place a value on projected results of behaviors [34]. If engaging in an eHealth intervention is not perceived valuable, people will not prioritize it. This intertwines our findings that when participants perceived the content irrelevant to their needs and expectations, the intervention was not perceived as valuable as other matters. Our findings relate to a previous investigation withdrawal from a telehealth intervention, revealing that the most frequent reason for withdrawal was that the participants did not perceive any benefit in using the telehealth service (eg, submitting their blood glucose readings to staff in local monitoring centers) [37]. One explanation for the lack of perceived value of the intervention is that some participants in our study said they already controlled their diabetes well, that they did not consider themselves as sick, or did not want to focus too much on diabetes in their daily lives. More than half of the participants had acceptable levels of HbA_1c_ prior to start, reaching the expected treatment goal of ≤ 7%. This could explain why they did not perceive a need for the intervention. Another explanation could be that even though their nurse deemed them suitable candidates for the intervention, they themselves did not want to put diabetes “up front.” They were uncomfortable with, or regarded the issues raised in the reflection sheets as too demanding. Others preferred to focus on living their lives, not on the diabetes.

Patients’ perspective of “wellness-in-the foreground” has been addressed in the shifting perspectives model, describing that people with chronic illness varies their attention of their disease [38]. Complex lives and competing priorities are important factors for developers to consider when designing “real-world” eHealth interventions for diabetes self-management support, to create successful engagement strategies and approaches that are likely to reach and engage the target population.

Some participants did not see the relevance of the structured reflection sheets in the GSD eHealth intervention as relevant to them. This matter relates to the discussion of the consequences for motivation when an activity is not perceived as valuable enough and could indicate that the current intervention, with its complex aspects and delivery method, is not suitable for all participants. These findings can have two possible explanations. First, the reflection sheets address aspects of people’s lives and emotions which may differ from what the participants are accustomed to and what they expect from communication with their nurse. The patients are asked to reflect on their challenges and make a plan for ideal problem solving (Table 1), which may differ from the traditional health care for people with diabetes, which are more concerned with education and information [7]. As the approach differs, it seems important to provide potential participants tailored information about the aim, the content, and the effort needed to remain engaged in the GSD intervention in order to recruit eligible participants who want to take part in and value such an intervention. Second, filling out reflection sheets electronically and communicating in writing could affect participants’ perception of the purpose and value of the questions. The intervention aims to support each individual’s autonomous goal setting and action planning [23], which are key features in self-management support interventions for people with diabetes. However, it was designed for face-to-face meetings. Perhaps the issues raised in the reflection sheets are so complicated that some participants would benefit from verbal explanation and discussion.

#### Technology

Previous research addresses technical problems as a continuous challenge in eHealth interventions resulting in high dropout rates [17,39]. Intelligible and user-friendly technology is imperative to maintain engagement and achieve benefits from digital health interventions [40]. Our findings concerning frustrating technology may therefore not be surprising. However, it is still important to address this issue, as most of our participants described difficulty with the technological solution. This finding may reflect that the demand for security level 4 (see Textbox 1) on patient-provider communication solutions is a barrier to engagement in such interventions. In addition, conducting the intervention depended on participants being able to download and upload PDFs to secure messages, which many participants found cumbersome. Our findings thus indicate that the eHealth technology offered in this study was not sufficiently user-friendly. Earlier research exploring patients’ experiences with a diabetes self-management portal reveals technical challenges such as slow Internet access and time-consuming and difficult data entry as barriers to use. Improving the convenience of Web portals seems important to improve usability and reduce attrition [41]. Our findings add to this evidence, indicating that there is still a large potential for improvement in eHealth product design to ensure technology that patients will engage in and use. The frustrating technology may have thwarted the participants’ sense of competence in managing the Web solution, and thus, reduced their engagement with the intervention. This points to the importance of facilitating more user-friendly but high security-level eHealth technology that would support users’ sense of competence in managing the solution, and thus, increase their autonomous motivation for intervention engagement. However, experiencing a sense of competence supports autonomous motivation only when accompanied by self-determination [42]. This underlines the importance of creating successful engagement strategies and developing approaches that are likely to reach and engage the target population that can identify with or place a value on the projected results of engagement in the intervention.

### Strengths and Limitations

The findings from this study may serve as a basis for future research aimed at broadening our understanding of the dynamics of withdrawing from eHealth interventions. However, generalizations from this small and situational study are not possible, nor are they intended. Out of 13 participants who dropped out of the intervention, 12 agreed to be interviewed. Although this could be considered a small sample, it is a strength of this study that most of the participants who dropped out were willing to be interviewed. The semistructured interview guide allowed the participants to express their genuine experiences, providing rich data. As the interviewer had no relationship with the participants, the participants might have felt more comfortable being candid. However, we cannot rule out the possibility that the nuances of face-to-face interaction are lost so that misleading information may not be detected [30]. Moreover, to reinforce the credibility of the data collection, the same researcher conducted all interviews. The findings and interpretations were discussed by a group of researchers, which also reinforced the credibility of the analysis.

A limitation that should be mentioned was the uneven gender distribution of the participants in this study. Initially, 14 men and 4 women were included, of which only 10 men and 2 women were interviewed. In relative terms, more men than women withdrew from the intervention. eHealth interventions may be used and experienced differently by men and women. A systematic literature review argues that there are gender differences in needs, preferences, and Web-based communication styles when engaging in Web-based health communication [43]. The dropout rate and the results of this study might have been different had we been able to include more women in the intervention. However, as this is a small sample, these are only speculations, and we cannot draw any definitive conclusions. Another limitation was interviewing only participants. Data from the study nurses about their experiences of conducting the intervention and their explanations concerning why patients left the intervention could have introduced other perspectives and improved our understanding of why some participants withdrew from the intervention.

### Conclusions

Our findings indicate that the eHealth intervention based on GSD without face-to-face encounters with nurses reduced participants’ motivation for engagement in the intervention. To maintain motivation, our study points to the importance of combining eHealth with regular face-to-face consultations. Our study also shows that the perceived benefit of the GSD eHealth intervention intertwined with choosing to focus on other matters in complex daily lives are critical aspects in motivation for such interventions. This indicates the importance of giving potential participants tailored information about the aim, the content, and the effort needed to remain engaged in complex intervention so that eligible participants are recruited. Finally, motivation for engagement in the eHealth intervention was influenced by the technology used in this study. It seems important to facilitate more user-friendly but high-security eHealth technology. Our findings have implications for improving the eHealth intervention and to inform researchers and health care providers who are organizing eHealth interventions focusing on self-management support, in order to reduce dropout rates.

## Abbreviations

GPs: general practitioners
GSD: Guided Self-Determination
HbA1c: glycosylated hemoglobin
PDF: portable document format
SDT: self-determination theory
T2DM: type 2 diabetes mellitus
